# Effect of a Telemedicine Model on Patients With Heart Failure With Reduced Ejection Fraction in a Resource-Limited Setting in Vietnam: Cohort Study

**DOI:** 10.2196/67228

**Published:** 2025-03-19

**Authors:** Hoai Thi Thu Nguyen, Hieu Ba Tran, Phuong Minh Tran, Hung Manh Pham, Co Xuan Dao, Thanh Ngoc Le, Loi Doan Do, Ha Quoc Nguyen, Thom Thi Vu, James Kirkpatrick, Christopher Reid, Dung Viet Nguyen

**Affiliations:** 1 Department of Cardiology Faculty of Medicine University of Medicine and Pharmacy, Vietnam National University, Hanoi Hanoi Vietnam; 2 Vietnam National Heart Institute Bach Mai Hospital Hanoi Vietnam; 3 Department of Cardiology Hanoi Medical University Hanoi Vietnam; 4 Bach Mai Hospital Hanoi Vietnam; 5 University of Medicine and Pharmacy, Vietnam National University, Hanoi Hanoi Vietnam; 6 Department of Science and Technology Hanoi People's Committee Hanoi Vietnam; 7 Division of Cardiology Department of Medicine University of Washington Medical Center Seattle, WA United States; 8 School of Population Health Curtin University Perth Australia; 9 School of Public Health and Preventive Medicine Monash University Melbourne Australia

**Keywords:** heart failure, HFrEF, telemedicine, telecare, remote monitoring, remote management, heart failure hospitalization, all-cause mortality, Vietnam, telehealth, heart, cardiology, cohort, remote, monitoring, resource-limited, cost-effective, low-cost, cardiovascular disease, hospitalization, mortality

## Abstract

**Background:**

Heart failure (HF) is a complex, life-threatening condition marked by high morbidity, mortality, reduced functional capacity, poor quality of life, and substantial health care costs. HF with reduced ejection fraction (HFrEF) represents the subgroup of HF with the highest risks of mortality and hospitalization, necessitating the prioritization of care and management models to optimize treatment outcomes in these patients. Currently, data on the effectiveness of telemedicine models in resource-limited settings, such as low- and middle-income countries, are scarce.

**Objective:**

This study aimed to evaluate the impact of telemedicine on improving prognosis in patients with HFrEF in Vietnam.

**Methods:**

In this prospective cohort study, we recruited patients who received either remote monitoring and management (telemedicine) or standard monitoring and management (usual care) in the outpatient department of the Vietnam National Heart Institute, Bach Mai Hospital, Hanoi, Vietnam. Eligible patients were ≥18 years old, had a diagnosis of HFrEF defined as left ventricular ejection fraction (LVEF) ≤40%, had a history of HF hospitalization within the past 12 months, and presented with clinical symptoms classified as New York Heart Association (NYHA) II or III. The primary composite outcome was defined as the time to the first unplanned HF hospitalization or all-cause mortality. The follow-up period for all outcomes extended to 12 months.

**Results:**

In total, 426 patients (298/426, 70% male; 128/426, 30% female) with a mean age of 61.3 (SD 14.6) years and a mean LVEF of 32.1% (SD 6.0%) were included in our study. Of these patients, 121 received telemedicine care, while 305 received usual care. The primary outcome occurred in 23 (23/121, 19%) patients in the telemedicine group and 82 (82/305, 26.9%) patients in the usual care group during the follow-up period, indicating a significant reduction in risk (adjusted hazard ratio [aHR] 0.57, 95% CI 0.35-0.94; *P*=.03). However, this effect was primarily driven by a significant reduction in unplanned HF hospital admissions (aHR 0.57, 95% CI 0.33-0.98; *P*=.04) rather than in all-cause mortality (aHR 0.77, 95% CI 0.36-1.63; *P*=.49).

**Conclusions:**

This study demonstrates that a simplified telemedicine model, even in resource-limited settings such as Vietnam, can effectively facilitate the remote monitoring and management of patients with HFrEF, resulting in significant reductions in HF-related hospitalizations and all-cause mortality.

**Trial Registration:**

National Agency for Science and Technology Information (NASATI), Vietnam CT07/01-2022-3; https://nsti.vista.gov.vn/projects/dth/xay-dung-mo-hinh-theo-doi-va-tu-van-suc-khoe-tim-mach-tu-xa-tai-thanh-pho-ha-noi-109276.html

## Introduction

The universal definition of heart failure (HF) describes it as a clinical syndrome characterized by symptoms or signs caused by structural or functional cardiac abnormalities, corroborated by elevated natriuretic peptide levels or objective evidence of pulmonary or systemic congestion [1,2]. HF has long been regarded as a global pandemic, with an estimated 64 million individuals worldwide with this condition, and this number continues to rise [3]. The economic burden of HF on global health care systems is substantial and continuously increasing due to the growing incidence of HF [3]. In the United States alone, the total cost of HF is projected to reach approximately US $70 billion by 2030 [4].

Despite significant advances in HF treatment over the past decades, mortality rates remain high, with 1-year mortality rates ranging from 14% to 30% and 5-year rates ranging from 50% to 75% [3,5]. Additionally, patients with HF frequently require hospitalization due to acute exacerbations of the disease, making HF the leading cause of hospitalization for individuals older than 65 years in Europe [2,5-7]. The frequency of hospitalizations is strongly associated with disease progression and increases the mortality risk in this patient population [2,5,7,8]. HF is classified based on left ventricular ejection fraction (LVEF) into 3 categories: HF with reduced LVEF (HFrEF), HF with mildly reduced LVEF, and HF with preserved LVEF, with LVEF values of ≤40%, 41% to 49%, and ≥50%, respectively [2,8]. HFrEF accounts for approximately one-half of all patients with HF and has the highest mortality and hospitalization rates among the 3 groups [2,5]. Furthermore, guided by abundant evidence synthesized from landmark trials, the management of HFrEF based on guideline-directed medical therapy recommended by leading cardiovascular societies promises significant benefits for this patient group [2,6]. In contrast, most clinical trials have failed to identify truly effective therapies for patients with HF with mildly reduced LVEF and HF with preserved LVEF, resulting in relatively limited and low-grade evidence in the treatment recommendations for these HF subtypes. Given this context, in resource-limited settings such as Vietnam, it is essential to prioritize the development of strategies, such as telemedicine models, to optimize the care and management of high-risk patients, particularly those with HFrEF, who are more likely to derive substantial benefits from proper monitoring and care. This approach represents a more resource-efficient strategy than focusing on the other 2 HF subtypes.

Over the past decade, remote monitoring and telemedicine solutions have emerged as promising strategies to enhance HF care and management by enabling closer patient monitoring and facilitating timely, personalized clinical decision-making by health care providers [7,9,10]. Numerous clinical trials have demonstrated the benefits of telemedicine for patients with HF, particularly for reducing the risk of HF-related hospitalizations [9-12]. However, most of these studies have been conducted in high-income countries using advanced patient monitoring technologies in telemedicine models. Conversely, data on the benefits of telemedicine in low- and middle-income countries (LMIC) like Vietnam, where HF is rapidly becoming a significant health care burden, remain limited [13].

Against this backdrop, we conducted this study to evaluate the effectiveness of a simplified telemedicine model designed to accommodate the limited resources available in LMIC at improving the prognosis of patients with HFrEF in Vietnam.

## Methods

### Study Design, Setting, and Participants

This prospective cohort study was conducted at the Vietnam National Heart Institute (VNHI), Bach Mai Hospital, Hanoi, Vietnam. Between January 2023 and October 2023, the study recruited 2 cohorts of patients managed and monitored in the outpatient department at VNHI. One cohort consisted of patients who received remote monitoring and management (telemedicine model), while the second cohort received standard monitoring and management (usual care model). This study was registered with the National Agency for Science and Technology Information, Vietnam (registration number: CT07/01-2022-3). As the first study to evaluate the effectiveness of a telemedicine model for patients with HF in Vietnam, the findings and experiences obtained during the implementation of this telemedicine program will provide a foundation for refining the model and expanding its application to other health care facilities across the country. Bach Mai Hospital was selected as the sole study site because it is the only center classified as the highest level in northern Vietnam’s hierarchical health care system. Geographically, its location in Hanoi, the capital city and central hub of northern Vietnam, facilitates access to patients from diverse regions across the north. Furthermore, with a team of highly experienced physicians and nurses specializing in HF management and medical education, the implementation of the telemedicine model at Bach Mai Hospital was expected to not only ensure the highest quality of care for patients enrolled in this study but also enhance the potential for successful knowledge and technique transfer to lower-level health care facilities in the future.

Participants were eligible if they (1) were aged 18 years or older, (2) were diagnosed with HF according to European Society of Cardiology criteria with an LVEF ≤40% [2], (3) had been admitted to the hospital for acute HF within 12 months prior to starting the study, and (4) had a clinical presentation of NYHA classification of II or III. Patients were excluded from the study if they had severe mental or physical disorders (eg, major depression), were undergoing hemodialysis, or had been admitted to the hospital for any reason within the previous week. Additionally, the exclusion criteria included patients with a left ventricular assist device or those who had experienced a pulmonary embolism, acute stroke, cardiogenic shock, cardiac surgery, coronary revascularization, or cardiac resynchronization therapy implantation within 1 month prior to the study. Patients scheduled for coronary revascularization, transcatheter aortic valve implantation, transcatheter mitral valve repair, or cardiac resynchronization therapy implantation within the next 3 months were also excluded. Furthermore, patients with severe pulmonary diseases, including chronic obstructive pulmonary disease (stage C/D), uncontrolled asthma, or pulmonary hypertension (World Health Organization [WHO] class III-IV), as well as pregnant women, were not eligible for inclusion. In our study, the necessary costs (for patient management and provision of measurement devices) for patients in the telemedicine group were significantly higher than the costs for patients in the usual care group (where care and management continue as usual, with coverage by insurance or out-of-pocket payment by patients). To optimize the research resources, we chose a ratio of 1:2.5 rather than a balanced patient ratio of 1:1 between the 2 groups. This approach helped reduce the number of patients required in the telemedicine group, thus lowering the total costs while still ensuring sufficient statistical power. Using a sample size calculation for a time-to-event end point with a maximum follow-up period of 12 months and assuming median survival times of 17 months for the usual care group and 29 months for the treatment group, with a significance level of .05 and patient ratio of 1:2.5 between the telemedicine and usual care groups, the minimum required sample size was calculated to be 120 patients for the telemedicine group and 298 patients for the usual care group to achieve 80% power [9,14]. To account for potential withdrawals, an additional 3.5% was added, resulting in a final estimated sample size of 433 patients.

### Study Procedures

The remote monitoring and management program for outpatients, referred to as the telemedicine model, was approved and implemented in the outpatient department at VNHI, Bach Mai Hospital, beginning in January 2023. Due to the unavailability of advanced specialized monitoring devices in Vietnam, our center developed a telemedicine model using simple monitoring devices that patients and caregivers can easily operate at home, such as automatic electronic blood pressure monitors and weighing scales. Throughout the study period, all potential patients seen and managed in the outpatient department were assessed by their primary treating physicians and invited to participate in the telemedicine program.

Our recruitment process consisted of 2 phases. In Phase 1, all patients meeting the inclusion criteria and consenting to participate in the study were assigned to the telemedicine group. Patients who declined participation in the telemedicine group were assigned to the usual care group. This process continued until the target number of patients for the telemedicine group (n=123) was reached. In Phase 2, all patients meeting the inclusion criteria and consenting to participate in the study were allocated to the usual care group. This phase continued until the target number of patients for the usual care group (n=310) was achieved.

Upon enrollment in the telemedicine program, patients were required to report their health status to the telemedicine center daily. The information provided included blood pressure, heart rate, and body weight measurements taken at home using validated devices (either by the patients themselves or their caregivers), as well as any new or unusual symptoms such as dyspnea, fatigue, chest pain, palpitations, swelling, cough, or fever. This reporting followed the Self-Check Plan for HF management recommended by the American Heart Association [6]. Reporting of information was encouraged to be conducted via a specialized app developed for our telemedicine program, accessible on smartphones, either by the patients themselves or their caregivers. Symptoms were entered as a checklist, while measurement results were input into designated fields, and any specific signs were reported as free-text entries. The app was designed to validate the completeness of the entered information and display warnings if essential data were missing. Additionally, the app issued daily reminders at 8 PM or a time selected by the patient, alerting them to submit their health information to the telemedicine center if they had not already done so. Information collected through this method was automatically stored in the patient’s profile within the telemedicine system database. For patients without access to smartphones or those experiencing difficulties using the app, alternative reporting methods were available. Patients or caregivers could report information via SMS text messaging (including photos of measurement results displayed on devices) or through direct video or phone calls to telemedicine center nurses. In such cases, the nurses manually entered the reported information into the system database. At 8 PM daily, or at the patient’s chosen time, if no health updates had been received, the system sent alerts to the telemedicine center staff, prompting them to proactively contact the patient to collect the necessary information. Patient data were securely stored in the telemedicine center’s online database, accessible at any time by cardiologists on our clinical team.

Based on the daily information provided by patients and the health history recorded in the database, nurses at the telemedicine center assessed whether a more detailed evaluation by a specialist was necessary. The following signs or symptoms (either newly developed or pre-existing but worsening during this episode) were considered indicative of an unstable condition with a high risk of acute decompensated HF, thereby necessitating a review of the patient’s health status by a cardiologist: frequent cough, shortness of breath at rest, inability to lie flat due to dyspnea, confusion, dizziness, loss of appetite, greater difficulty sleeping, sadness or depression, increased swelling in the lower extremities, sudden weight gain of more than 1 kg within 24 hours or 2 kg in a week, systolic blood pressure (SBP) <90 mm Hg, diastolic blood pressure (DBP) <60 mm Hg, heart rate >100 bpm, blood pressure >180/110 mm Hg, or SBP or DBP increase or decrease >20 mm Hg compared with the average SBP or DBP of the previous day. In certain cases, an additional real-time interview with patients was arranged to collect further information. After gathering all necessary information from the patient’s online medical records and direct interviews, physicians were granted autonomy to make decisions based on their clinical judgment and the European Society of Cardiology guidelines for the diagnosis and management of acute HF [2]. We did not propose a rigid protocol for physicians within this telemedicine model, as flexibility was deemed essential given the variability in patients’ clinical conditions, geographic differences, and the availability of health care services in their respective areas. Physicians’ decisions may include advising the patient to visit the emergency department, seek hospitalization at a cardiology center, or consult a local clinic for further evaluation (eg, electrocardiography, chest X-ray, echocardiography, laboratory tests). Other options may involve adjusting medication (limited to oral drugs), recommending lifestyle changes or home exercises, or determining that no further action is required. In more complex cases, a multidisciplinary team at the telemedicine center would discuss the situation to reach a final decision. In addition to nurse-initiated evaluations, each patient’s health records were reviewed biweekly by telemedicine center physicians based on the online database.

The usual care group comprised patients who were monitored and managed through the standard process in the outpatient department at the VNHI. Throughout the study, these patients remained under the supervision of cardiologists and other physicians in the outpatient department. Treatment adjustments or prescriptions were based solely on the independent decisions of their primary treating doctors and were not influenced by the patient’s participation in the study. Follow-up visits were typically scheduled every 3 months but could vary depending on the patient’s condition, as assessed by the primary treating doctors.

Baseline data (including demographics; anthropometrics; clinical history; signs and symptoms; medication lists; and data from electrocardiograms, echocardiograms, and lab tests) were collected at the time of patient enrollment. BMI was classified according to the WHO diagnostic criteria for Asian adults: A BMI of 18.5-22.9 kg/m² was considered as normal weight, <18.5 kg/m² as underweight, 23-24.9 kg/m² as overweight, and ≥25 kg/m² as obese [15]. We did not use medication adherence of patients as an outcome of interest due to concerns that missing data from patients who died or were lost to follow-up could introduce significant bias.

### Study Outcomes

The primary outcome of the study was defined as a composite end point consisting of the first unplanned hospitalization due to HF or all-cause mortality, evaluated over a follow-up period of up to 12 months after patient enrollment. Secondary outcomes included (1) all-cause mortality and (2) the first unplanned hospitalization specifically due to HF.

### Statistical Analysis

Baseline characteristics were summarized as mean (SD) or median (IQR), as appropriate, for continuous variables and as numbers (percentages) for categorical variables. The between-group differences in continuous variables were tested using either the independent Student *t* test or the Wilcoxon rank-sum test, depending on their distribution. Categorical variables were compared using Pearson chi-square tests or Fisher exact tests, as appropriate.

All survival analyses of outcomes were conducted on a time-to-first-event basis. Cumulative incidence curves for all-cause mortality were generated using the Kaplan-Meier method to visually represent survival probabilities over time. Differences between the survival curves of the 2 groups were evaluated using the log-rank test to determine statistical significance. To assess the impact of telemedicine on the primary and secondary outcomes, multivariable Cox-proportional hazards regression models were applied, estimating hazard ratios (HRs) and the corresponding 95% CIs while adjusting for potential confounders. The confounding variables incorporated into the multivariable analysis were determined by a panel of HF experts from the Vietnam Heart Association. These variables were selected based on well-documented predictors of mortality and HF hospitalization in patients with HFrEF, as outlined in clinical guidelines and supported by previous studies [1,2,6,8,13,16]. Specifically, the variables included baseline demographic characteristics (age, sex), BMI classification, comorbidities, LVEF, the use of guideline-directed medical therapy for HFrEF, and N-terminal pro-B-type natriuretic peptide (NT-proBNP) levels. In addition, these variables, as well as the analyses of the primary composite end point and secondary end points (mortality and HF hospitalization), were prespecified before the commencement of the study. Furthermore, we conducted a sensitivity analysis, incorporating additional variables (cardiac comorbidities, relevant medications, socioeconomic status, and health literacy–related factors) into the multivariable Cox regression models to validate the robustness of the results from the primary analyses. Event rates were reported as the number of events per 100 patient-years of follow-up, accounting for any censored data due to loss to follow-up or the end of the study period.

All statistical analyses were performed using Stata SE version 18.0. All comparisons were 2-tailed, and *P* values <.05 were considered statistically significant.

### Ethical Considerations

The study was conducted in accordance with the principles of the Declaration of Helsinki. The study protocol was reviewed and approved by the Institutional Review Board of Bach Mai Hospital, Hanoi, Vietnam (number: 1973/BM-HĐĐĐ). All participants provided written informed consent prior to enrollment, after receiving a comprehensive explanation of the study’s purpose, procedures, and potential risks and benefits. To protect participant privacy, all personal identifiers were removed from the data set.

## Results

### Participants

A total of 426 patients with HFrEF (298/426, 70% male; mean age 61.3, SD 14.6 years), including 121 patients in the telemedicine group (81/121, 66.9% male; mean age 60.6, SD 15.4 years) and 305 patients in the usual care group (217/305, 71.2% male; mean age 61.6, SD 14.4 years), were involved in this study (Multimedia Appendix 1). Of the 273 eligible patients who initially declined participation in the study, 187 declined because they did not plan to continue HF management at Bach Mai Hospital, 41 declined due to concerns about the safety of their health during participation, 29 declined due to concerns about the additional time or costs associated with participation, and 16 declined due to concerns regarding the security of their privacy and personal information. At the end of Phase 1 of the recruitment process, a total of 123 patients were recruited into the telemedicine group, while 15 patients were assigned to the usual care group. A survey identified 2 primary reasons for patients agreeing to participate in the study but refusing to join the telemedicine group: (1) concerns about the treatment efficacy of the telemedicine model, as it was a new approach not previously implemented in Vietnam (13/15, 87%), and (2) concerns about potential additional costs associated with participation in the telemedicine model (2/15, 13%). In Phase 2, an additional 295 patients were recruited into the usual care group to reach the target number of 310. Notably, among the 15 participants who opted out of the telemedicine intervention, 11 (73%) resided within a 5-km radius of our hospital, whereas only 42 (34%) of the 123 participants included in the telemedicine group lived within this proximity. This proximity, which facilitates regular follow-up visits at the hospital, may have contributed to the participants’ reluctance to choose telemedicine, a recently implemented health care model in Vietnam. The majority of patients were married (401/426, 94.1%) and resided in rural areas (259/426, 60.8%). Most reported a monthly income of US $300 to US $450 (275/426, 64.6%), slightly above the national average of US $300. Notably, all patients had health insurance (426/426, 100%), largely attributable to Vietnam’s universal health care coverage policy. In Table 1, we present the detailed baseline characteristics of the participants in the 2 groups. The mean LVEF of the overall population was 32.1% (SD 6.0%) and comparable between the telemedicine group (32.1%, SD 6.0%) and the usual care group (32.1%, SD 6.0%; *P*=.56).

The most common comorbidities among the study participants were hypertension (128/426, 30.1%), coronary artery disease (109/426, 25.6%), chronic kidney disease (93/426, 21.8%), and diabetes (91/426, 21.4%). The mean BMI of the study population was 22.1 (SD 2.9) kg/m^2^, with an obesity rate of 15.3% (65/426), based on the WHO classification for Asians, and an underweight rate of 10.6% (45/426). At the start of the study, 52.4% (223/426) of patients were being treated with an angiotensin-neprilysin inhibitor, 22.3% (95/426) with an angiotensin-converting enzyme inhibitor or angiotensin receptor blocker, 80.8% (344/426) with a sodium-glucose cotransporter-2 inhibitor, 70% (209/426) with beta-blockers, 68.5% (292/426) with a mineralocorticoid receptor antagonist (only spironolactone), 61.7% (263/426) with loop diuretics, and 7% (30/426) with thiazide-like diuretics (no patients were prescribed thiazide diuretics). Analysis results indicated no statistically significant differences in demographic characteristics, BMI, echocardiographic findings, laboratory measurements, comorbidities, socioeconomic status, health literacy, or therapeutic regimens between the telemedicine and usual care groups.

During the follow-up period, data from our telemedicine database indicated that, among the 121 patients in the telemedicine group, 75 patients (62%) reported a complete set of daily measurements on at least 90% of the total days enrolled in the telemedicine program. Additionally, 30 patients (30/121, 25.6%) provided complete measurements on 70% to 90% of the days, 13 patients (13/121, 9.9%) on 50% to 70% of the days, and only 3 patients (3/121, 2.5%) on fewer than 50% of the days.

**Table 1:** Baseline characteristics of study participants.

Characteristic			Total sample (n=426)		Usual care (n=305)		Telemedicine (n=121)		*P* value	
Age (years), mean (SD)			61.3 (14.6)		61.6 (14.4)		60.6 (15.4)		.50	
Gender (male), n (%)			298 (70)		217 (71.2)		81 (66.9)		.39	
BMI (kg/m^2^), mean (SD)			22.1 (2.9)		22.1 (2.9)		22.2 (2.8)		.74	
**BMI classifications, n** **(** **%)**										.88
	Underweight	45 (10.6)		32 (10.5)		13 (10.7)				
	Normal weight	232 (54.5)		165 (54.1)		67 (55.4)				
	Overweight	84 (19.7)		63 (20.7)		21 (17.4)				
	Obesity	65 (15.3)		45 (14.8)		20 (16.5)				
**Locality**										.73
	Rural	259 (60.8)		187 (61.3)		72 (59.5)				
	Urban	167 (39.2)		118 (38.7)		49 (40.5)				
**Marital status**										.60
	Never married	10 (2.4)		6 (2)		4 (3.3)				
	Married	401 (94.1)		287 (94.1)		114 (94.2)				
	Widowed/divorced	15 (3.5)		12 (3.9)		3 (2.5)				
**Education**										.99
	School	299 (70.2)		214 (70.2)		85 (70.3)				
	College/university	127 (29.8)		91 (29.8)		36 (29.8)				
**Monthly income (US $), n (%)**										.80
	High (>450)	151 (35.5)		107 (35.1)		44 (36.4)				
	Medium (300-450)	275 (64.6)		198 (64.9)		77 (63.6)				
**Monthly treatment affordability (US $), n (%)**										.80
	High (>125)	172 (40.4)		122 (40)		50 (41.3)				
	Medium (75-125)	254 (59.6)		183 (60)		71 (58.7)				
Health insurance (yes), n (%)			426 (100)		305 (100)		121 (100)		>.99	
**Comorbidities** **,** **n** **(** **%)**										
	Hypertension	128 (30.1)		85 (27.9)		43 (35.5)		.12		
	Diabetes	91 (21.4)		64 (21.0)		27 (22.3)		.76		
	Coronary artery disease	109 (25.6)		81 (26.6)		28 (23.1)		.47		
	Chronic kidney disease	93 (21.8)		69 (22.6)		24 (19.8)		.53		
	Previous stroke	25 (5.9)		20 (6.6)		5 (4.1)		.34		
	Atrial fibrillation	54 (12.7)		37 (12.1)		17 (14.1)		.59		
	Documented VT^a^	42 (9.9)		30 (9.8)		12 (9.9)		.98		
	Diagnosed VHD^b^	188 (44.1)		134 (43.9)		54 (44.6)		.90		
	Diagnosed CM^c^	162 (38.0)		119 (39.0)		43 (35.5)		.51		
**Pharmacotherapy** **,** **n** **(** **%)**										
	ARNI^d^	223 (52.4)		163 (53.4)		60 (50)		.47		
	ACEIs^e^/ARBs^f^	95 (22.3)		70 (23)		25 (20.7)		.61		
	SGLT2^g^ inhibitors	344 (80.8)		247 (81)		97 (80.2)		.85		
	Beta-blockers	298 (70)		217 (71.2)		81 (66.9)		.39		
	MRAs^h^	292 (68.5)		210 (68.9)		82 (67.8)		.83		
	Loop diuretics	263 (61.7)		184 (60.3)		79 (65.3)		.34		
	Thiazide-like diuretics	30 (7)		20 (6.6)		10 (8.3)		.54		
	Ivabradine	90 (21.1)		67 (22)		23 (19)		.50		
**Echocardiography findings, mean (SD)**										
	LVEF^i^ (%)	32.1 (6.0)		32.0 (6.0)		32.4 (6.2)		.56		
	PAP^j^ (mm Hg)	38.0 (11.3)		37.8 (10.5)		38.4 (12.9)		.64		
	TAPSE^k^ (mm)	19.2 (2.7)		19.2 (2.7)		19.2 (2.9)		.96		
	FAC^l^ (%)	38.9 (4.8)		38.7 (4.8)		39.3 (4.8)		.30		
	LAVi^m^ (mL/m^2^)	54.0 (26.2)		55.2 (26.9)		50.8 (24.1)		.12		
**Laboratory measurements** **, median (IQR)**										
	NT-proBNP^n^ (pmol/L)	1064 (807-1436)		1081 (818-1458)		1024 (749-1405)		.41		
	Serum potassium (mmol/L)	4.2 (4.0-4.5)		4.2 (4.0-4.5)		4.1 (3.9-4.4)		.10		
	Serum sodium (mmol/L)	138 (136-139)		138 (136-139)		138 (136-139)		.61		
	Haemoglobin (g/L)	141 (129-153)		141 (128-154)		140 (131-151)		.94		
	eGFR^o^ (mL/min/1.73m^2^)	53.2 (37.4-68.7)		51.9 (36.8-67.2)		56.7 (40.5-72.8)		.13		

^a^VT: ventricular tachycardia.

^b^VHD: valvular heart disease.

^c^CM: cardiomyopathy.

^d^ARNI: angiotensin-neprilysin inhibitor.

^e^ACEIs: angiotensin-converting enzyme inhibitors.

^f^ARBs: angiotensin receptor blockers.

^g^SGLT2: sodium-glucose cotransporter-2.

^h^MRAs: mineralocorticoid receptor antagonists.

^i^LVEF: left ventricular ejection fraction.

^j^PAP: pulmonary artery pressure.

^k^TAPSE: tricuspid annular plane systolic excursion.

^l^FAC: fractional area change.

^m^LAVi: left atrial volume index.

^n^NT-proBNP: N-terminal pro-B-type natriuretic peptide.

^o^eGFR: estimated glomerular filtration rate.

### Primary Outcome

**Figure 1:**
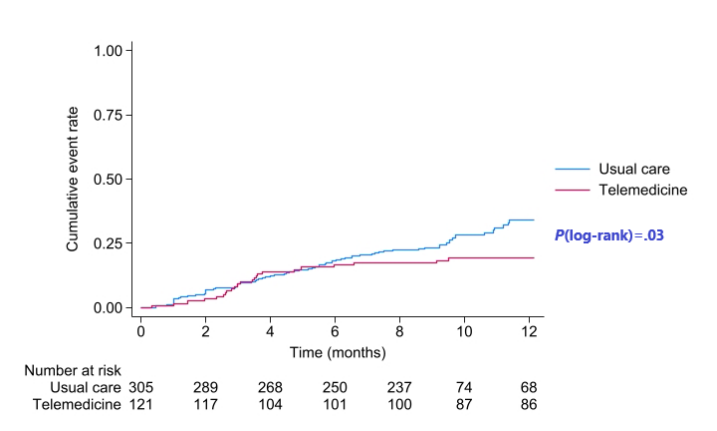
Kaplan-Meier cumulative event curve for the primary composite outcome.

During the 12-month follow-up period, the primary end point occurred in 23 of 121 patients (19%) in the telemedicine group and in 82 of 305 patients (26.9%) in the usual care group. Specifically, there were 10 deaths (10/121, 8.3%) and 19 first unplanned HF hospitalizations (19/121, 15.7%) in the telemedicine group, compared with 29 deaths (29/305, 9.5%) and 71 first unplanned HF hospitalizations (71/305, 23.3%) in the usual care group. This indicates a significant reduction in the risk of the composite end point (first unplanned HF hospitalization or all-cause mortality) over the 12-month follow-up in the telemedicine group (adjusted HR 0.57, 95% CI 0.35-0.94; *P*=.03; Figure 1 and Table 2).

**Table 2:** Multivariable Cox proportional hazards regression assessing the effect of telemedicine on outcomes.

Variables				Primary outcome				All-cause mortality					Heart failure hospitalization		
				aHR^a^ (95% CI)		*P* value		aHR (95% CI)		*P* value		aHR (95% CI)			*P* value
**Care model**															
	Telemedicine		0.57 (0.35-0.94)		.03		0.77 (0.36-1.63)		.49		0.57 (0.33-0.98)			.04	
	Usual care		1.0 (reference)		—^b^		1.0 (reference)		—		1.0 (reference)			—	
Age (years)				1.01 (1.00-1.03)		.11		1.02 (1.00-1.05)		.10		1.02 (1.00-1.04)			.06
**Sex**															
	Male		1.26 (0.79-2.02)		.34		0.99 (0.49-2.02)		.98		1.37 (0.81-2.30)			.24	
	Female		1.0 (reference)		—		1.0 (reference)		—		1.0 (reference)			—	
LVEF^c^ (%)				0.92 (0.89-0.96)		<.001		0.98 (0.92-1.04)		.45		0.91 (0.88-0.95)			<.001
**BMI classification**															
	Underweight		1.10 (0.58-2.08)		.77		1.19 (0.47-3.03)		.72		1.06 (0.52-2.14)			.87	
	Normal weight		1.0 (reference)		—		1.0 (reference)		—		1.0 (reference)			—	
	Overweight		0.82 (0.46-1.45)		.49		0.77 (0.28-2.10)		.61		0.93 (0.51-1.70)			.81	
	Obese		1.36 (0.76-2.41)		.30		1.19 (0.43-3.30)		.73		1.44 (0.76-2.72)			.26	
Hypertension				0.65 (0.39-1.07)		.09		0.40 (0.16-1.00)		.051		0.76 (0.45-1.29)			.31
Diabetes				1.87 (1.17-2.98)		.009		1.04 (0.45-2.39)		.93		1.90 (1.16-3.14)			.01
CAD^d^				1.15 (0.73-1.84)		.54		0.93 (0.42-2.08)		.87		1.44 (0.89-2.34)			.14
CKD^e^				1.64 (0.99-2.72)		.056		1.78 (0.79-3.98)		.16		1.90 (1.11-3.25)			.02
Prior stroke				1.96 (0.97-3.93)		.06		3.38 (1.16-9.86)		.03		1.67 (0.77-3.63)			.19
Atrial fibrillation				1.08 (0.59-1.97)		.80		1.87 (0.80-4.35)		.15		1.10 (0.57-2.10)			.78
NT-proBNP^f^ (per 200 ng/mL)				1.00 (1.00-1.01)		.37		1.00 (0.99-1.01)		.99		1.00 (1.00-1.01)			.29
**Medication use**															
	**ARNI ^g^** **/ACEI^h^** **/ARB^i^**														
		Not used	1.0 (reference)		—		1.0 (reference)		—		1.0 (reference)			—	
		ACEIs/ARBs	1.82 (0.91-3.63)		.09		1.35 (0.42-4.37)		.62		1.55 (0.74-3.23)			.24	
		ARNI	1.83 (0.87-3.83)		.22		1.84 (0.55-6.13)		.32		1.73 (0.79-3.77)			.17	
	SGLT2^j^ inhibitors		0.97 (0.56-1.69)		.92		0.58 (0.24-1.41)		.23		1.09 (0.59-2.03)			.77	
	Beta-blockers		0.59 (0.38-0.92)		.02		0.46 (0.23-0.93)		.03		0.59 (0.36-0.95)			.03	
	MRAs^k^		0.78 (0.44-1.40)		.41		1.27 (0.47-3.45)		.63		0.82 (0.44-1.53)			.54	
	Loop diuretics		1.03 (0.60-1.75)		.92		1.25 (0.51-3.08)		.63		0.92 (0.53-1.62)			.78	

^a^aHR: adjusted hazard ratio.

^b^Not applicable.

^c^LVEF: left ventricular ejection fraction.

^d^CAD: coronary artery disease.

^e^CKD: chronic kidney disease.

^f^NT-proBNP: N-terminal pro b-type natriuretic peptide.

^g^ARNI: angiotensin–neprilysin inhibitor.

^h^ACEI: angiotensin-converting enzyme inhibitor.

^i^ARB: angiotensin receptor blocker.

^j^SGLT2: Sodium-glucose cotransporter-2.

^k^MRAs: mineralocorticoid receptor antagonist.

### Secondary Outcomes

**Figure 2:**
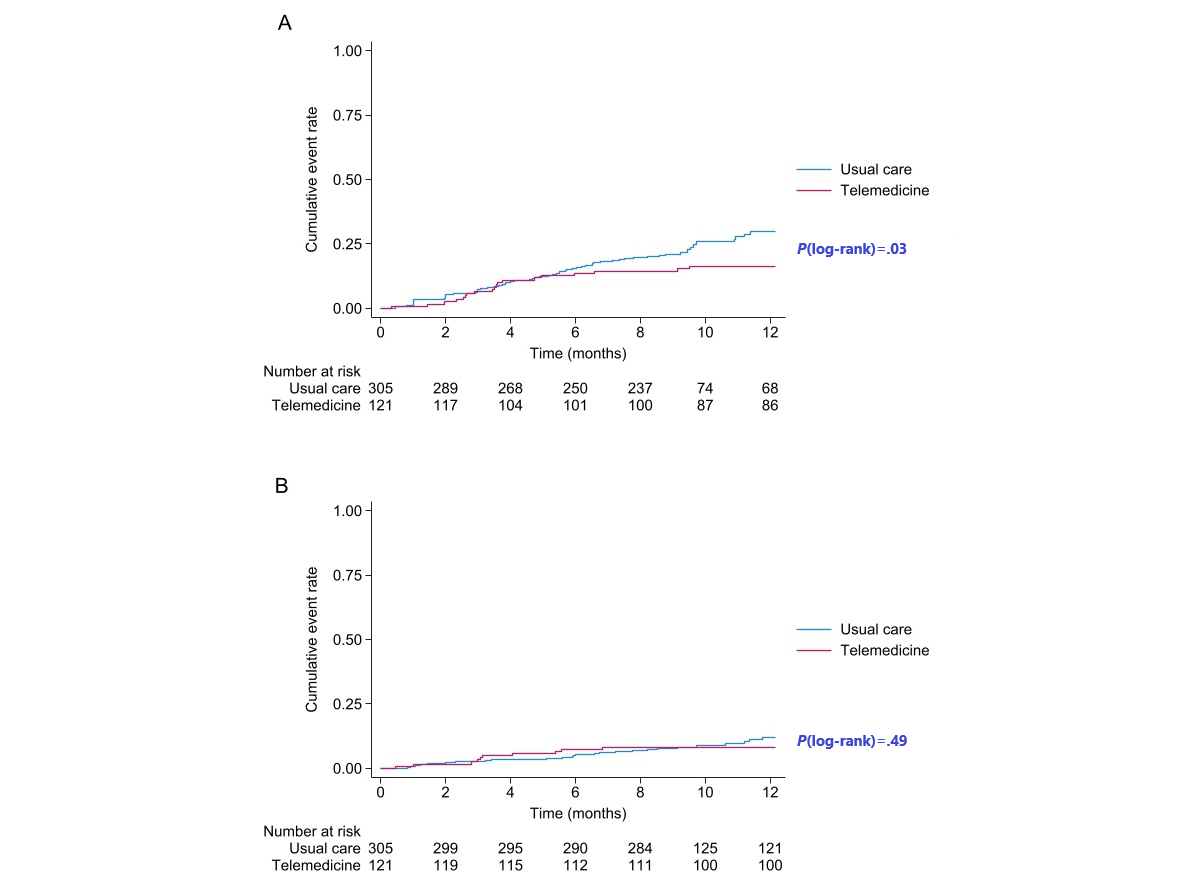
Kaplan-Meier cumulative event curve for the secondary outcomes: (A) first unplanned heart failure hospitalization, (B) all-cause mortality.

Regarding the secondary end points, after adjusting for covariables, telemedicine continued to demonstrate a significant reduction in the risk of HF hospitalization (adjusted HR 0.57, 95% CI 0.33-0.98; *P*=.04; Figure 2 and Table 2). Conversely, although there was a trend suggesting a slight reduction in mortality risk in the telemedicine group, the difference was not statistically significant (adjusted HR 0.77, 95% CI 0.36-1.63; *P*=.49; Figure 2 and Table 2).

### Sensitivity Analysis

The sensitivity analysis, incorporating additional variables (documented ventricular tachycardia, diagnosed valvular heart disease, diagnosed cardiomyopathy, thiazide-like use, ivabradine use, locality, marital status, education, monthly income, monthly treatment affordability) into the multivariable Cox regression models (Multimedia Appendix 2) for the outcomes of interest, demonstrated that the efficacy of telemedicine remained consistent with the primary analysis (Table 2), suggesting the robustness of the results.

## Discussion

### Principal Findings

To the best of our knowledge, this is the first study to evaluate the efficacy of telemedicine for reducing mortality and HF hospitalization among patients with HFrEF in Vietnam. In this prospective cohort study, we demonstrated that a resource-limited telemedicine model, which does not require the support of advanced specialized devices, could still provide significant prognostic benefits for HFrEF patients. Specifically, patients monitored and managed through telemedicine had a 43% lower risk of experiencing the primary outcome compared with those receiving usual care (adjusted HR 0.57, 95% CI 0.35-0.94; *P*=.03). However, this composite primary outcome was primarily driven by a significant reduction in unplanned HF hospital admissions (adjusted HR 0.57, 95% CI 0.33-0.98; *P*=.04) rather than in all-cause mortality (adjusted HR 0.77, 95% CI 0.36-1.63; *P*=.49).

### Comparison With Prior Work

Patients with HFrEF exhibit the highest rates of hospitalization and mortality among patients with HF [5,17,18]. Furthermore, there is a well-documented pattern of gradually increasing cardiac filling pressures, even during the “stable” phase following hospital discharge [19,20]. This progressive rise in congestion ultimately leads to the recurrence of symptoms and, consequently, the need for rehospitalization [16,21]. As a result, this population requires close monitoring and management to prevent and address exacerbations while simultaneously promoting patient self-management and empowerment. Remote monitoring and telemedicine have emerged as critical components in the management of HF, offering significant improvements in patient outcomes while optimizing health care resource allocation [7,22]. A primary benefit is the increased accessibility to care, facilitating real-time monitoring of physiological parameters and symptoms from patients’ homes [7,22]. This approach minimizes the necessity for frequent in-person clinic visits and promotes patient engagement in self-management, thereby reducing the logistical burden of travel [7]. Furthermore, evidence suggests that remote monitoring allows for the early detection of HF deterioration, enabling timely therapeutic adjustments and decreasing the risk of hospitalizations [23,24]. A meta-analysis of 29 randomized controlled trials (RCTs) published in 2019 demonstrated that telemedicine significantly improves quality of life and reduces the risk of HF-related hospitalization in patients with HF [25]. One notable RCT is the TIM-HF2 study conducted in Germany, which included 1571 patients with NYHA class II or III HF with an LVEF ≤45%, with a maximum follow-up duration of 393 days [9]. The telemedicine model used in this trial was a structured remote patient management intervention in which patients were monitored daily via a telemonitoring system that included a 3-channel electrocardiogram device, a device to measure blood pressure, a device to measure SpO_2_, and weighing scales. This telemonitoring system also facilitated the transmission of data from the patient’s home to the telemedical center, where specialized software classified patient risk based on preprogrammed algorithms. The subsequent actions taken by the telemedical center’s physicians were informed by the patient’s risk level and health data within the system. The results of the TIM-HF2 trial revealed that the remote patient management group had a significantly lower all-cause mortality rate compared with the usual care group (HR 0.70, 95% CI 0.50-0.96; *P*=.03), although there was no significant difference observed in cardiovascular mortality (HR 0.67, 95% CI 0.45-1.01; *P*=.056) [9]. More recently, the multicenter AMULET trial conducted in Poland with 605 patients with HF and an LVEF ≤49% was published in 2021 [10]. Patients in the intervention group (telecare) were monitored and managed under the AMULET model, which included regular consultations with nurses at ambulatory care points. Information collected after each visit, including HF signs and symptoms and measurements of vital parameters, showed that, after 12 months of follow-up, patients in the telecare group experienced a 31% lower rate of composite events (unplanned HF hospitalizations or cardiovascular deaths) compared with those receiving standard care (HR 0.69, 95% CI 0.48-0.99; *P*=.04) [10]. Notably, the implementation of AMULET telecare primarily reduced the risk of first hospitalizations due to HF (HR 0.64, 95% CI 0.41-0.99; *P*=.04) but did not significantly impact the risks of cardiovascular mortality (HR 1.03, 95% CI 0.54-1.98; *P*=.93) or all-cause mortality (HR 0.99, 95% CI 0.59-1.67; *P*=.98) [10].

Although direct comparisons between our study and others using different telemedicine models may be limited due to variations in research methodologies, study populations, and telemedicine processes (eg, monitoring technologies, interventions, and communication methods), the comparable benefits observed in our research underscore the viability of remote health care models adapted to resource-limited settings, such as Vietnam and other LMIC [10,22]. Despite the absence of advanced monitoring devices, our telemedicine model demonstrated good efficacy, likely due to 3 main factors. First, the model ensures that physicians can regularly monitor patients’ weight, cardiovascular symptoms, and vital signs, which are the most critical information needed for early assessment and timely advice [7]. This helps to improve the prevention of congestion and reduce the risk of HF-related hospitalizations. Second, our protocol allows for direct communication between physicians and patients when necessary. This ensures the accuracy and clarity of information exchanged and enhances patient trust and adherence to treatment [7]. Finally, our study population consisted of high-risk HF patients (mean age >60 years, mean LVEF approximately 30%, with a history of HF hospitalization in the past 12 months), which may explain the significant benefit observed from the telemedicine model [7,9,23].

In high-income countries, health care systems are typically well-developed and uniformly distributed, with both urban and rural areas having well-resourced health care centers capable of providing high-quality care for a wide range of diseases. In contrast, Vietnam and most LMIC operate under a hierarchical health care model due to limited resources. In this model, only a few medical centers and hospitals in major cities receive prioritized investments, allowing them to employ highly skilled medical staff and use advanced technologies to provide high-quality care and optimize outcomes for complex medical conditions. Health care centers in rural and remote areas often receive minimal resources and lack specialists due to lower income and limited career development opportunities in these regions [26]. Consequently, these facilities can only provide basic health care services and lack the capacity to manage complex conditions like HF effectively. The implementation of telemedicine in Vietnam and other developing countries holds significant potential to overcome these limitations [26,27]. For patients in remote or underserved areas or those who face challenges in accessing specialized centers, telemedicine provides an opportunity for regular management, monitoring, and consultation with experienced physicians. This approach could improve health care quality, reduce costs and travel time, and enhance convenience for patients [22]. However, widespread adoption of telemedicine for HF management in resource-limited settings faces challenges due to the high costs of advanced monitoring devices commonly used in developed countries. Given the resource constraints in Vietnam and other developing countries, we propose a cost-effective, scalable telemedicine model that uses phone-based monitoring and affordable devices to measure essential parameters for HF management, such as blood pressure, heart rate, and body weight. The findings of this study demonstrate that this low-cost telemedicine model can provide significant benefits for patients with HFrEF in Vietnam compared with usual care. This approach may represent a viable strategy for enhancing HF care in resource-limited settings.

### Strengths and Limitations

Although this study represents the first evaluation of telemedicine’s efficacy in managing patients with HF in Vietnam using a simplified model designed for resource-limited settings, several limitations warrant consideration. First, as this was not an RCT, residual confounding variables may persist despite multivariable adjustments. This limitation could introduce bias, emphasizing the need for a well-designed trial to validate our findings in the future. Second, the single-center design limits the generalizability of the findings. The results may not be applicable to other health care settings or populations, particularly in regions or countries with differing health care infrastructures. Additionally, the cohort’s sociodemographic and clinical characteristics likely reflect the specific health care context in Vietnam, including unique patterns of comorbidities, health care access, and cultural factors. This may reduce the external validity of the findings when applied to different settings.

### Conclusions

Our study demonstrates that a simplified telemedicine model, without the inclusion of advanced monitoring devices in resource-limited settings such as Vietnam, can still effectively monitor and manage patients with HFrEF remotely, yielding significant benefits for reducing the risk of composite outcomes. However, this effect is primarily attributed to the reduction in unplanned HF hospitalizations rather than mortality. Future studies with more robust designs conducted across diverse regions and populations are necessary to validate these findings.

## Appendix

Multimedia Appendix 1 Study Flow Chart.

Multimedia Appendix 2 Sensitivity Analysis.

## Abbreviations

DBP: diastolic blood pressure
HF: heart failure
HFrEF: heart failure with reduced ejection fraction
HR: hazard ratio
LMIC: low- and middle-income countries
LVEF: left ventricular ejection fraction
NT-proBNP: N-terminal pro-B-type natriuretic peptide
NYHA: New York Heart Association
RCT: randomized controlled trial
SBP: systolic blood pressure
VNHI: Vietnam National Heart Institute
WHO: World Health Organization

## Data Availability

The data sets generated and/or analyzed during this study are available from the corresponding author on reasonable request.

## References

[1] Bozkurt B, Coats AJ, Tsutsui H, Abdelhamid M, Adamopoulos S, Albert N, Anker SD, Atherton J, Böhm Michael, Butler J, Drazner MH, Felker GM, Filippatos G, Fonarow GC, Fiuzat M, Gomez-Mesa J, Heidenreich P, Imamura T, Januzzi J, Jankowska EA, Khazanie P, Kinugawa K, Lam CSP, Matsue Y, Metra M, Ohtani T, Francesco Piepoli M, Ponikowski P, Rosano GMC, Sakata Y, SeferoviĆ P, Starling RC, Teerlink JR, Vardeny O, Yamamoto K, Yancy C, Zhang J, Zieroth S (2021). Universal definition and classification of heart failure: a report of the Heart Failure Society of America, Heart Failure Association of the European Society of Cardiology, Japanese Heart Failure Society and Writing Committee of the Universal Definition of Heart Failure. J Card Fail.

[2] McDonagh TA, Metra M, Adamo M, Gardner RS, Baumbach A, Böhm Michael, Burri H, Butler J, Čelutkienė J, Chioncel O, Cleland JGF, Coats AJS, Crespo-Leiro MG, Farmakis D, Gilard M, Heymans S, Hoes AW, Jaarsma T, Jankowska EA, Lainscak M, Lam CSP, Lyon AR, McMurray JJV, Mebazaa A, Mindham R, Muneretto C, Francesco Piepoli M, Price S, Rosano GMC, Ruschitzka F, Kathrine Skibelund A, ESC Scientific Document Group (2021). 2021 ESC Guidelines for the diagnosis and treatment of acute and chronic heart failure. Eur Heart J.

[3] Savarese G, Becher PM, Lund LH, Seferovic P, Rosano GMC, Coats AJS (2023). Global burden of heart failure: a comprehensive and updated review of epidemiology. Cardiovasc Res.

[4] Virani SS, Alonso A, Aparicio HJ, Benjamin EJ, Bittencourt MS, Callaway CW, Carson AP, Chamberlain AM, Cheng S, Delling FN, Elkind MSV, Evenson KR, Ferguson JF, Gupta DK, Khan SS, Kissela BM, Knutson KL, Lee CD, Lewis TT, Liu J, Loop MS, Lutsey PL, Ma J, Mackey J, Martin SS, Matchar DB, Mussolino ME, Navaneethan SD, Perak AM, Roth GA, Samad Z, Satou GM, Schroeder EB, Shah SH, Shay CM, Stokes A, VanWagner LB, Wang N, Tsao CW, American Heart Association Council on Epidemiology and Prevention Statistics Committee and Stroke Statistics Subcommittee (2021). Heart disease and stroke statistics-2021 update: a report from the American Heart Association. Circulation.

[5] Shahim B, Kapelios CJ, Savarese G, Lund LH (2023). Global public health burden of heart failure: an updated review. Card Fail Rev.

[6] Writing Committee Members, ACC/AHA Joint Committee Members (2022). 2022 AHA/ACC/HFSA guideline for the management of heart failure. J Card Fail.

[7] Stevenson LW, Ross HJ, Rathman LD, Boehmer JP (2023). Remote monitoring for heart failure management at home. J Am Coll Cardiol.

[8] Nguyen DV, Le TN, Truong BQ, Nguyen HTT (2025). Efficacy and safety of angiotensin receptor-neprilysin inhibition in heart failure patients with end-stage kidney disease on maintenance dialysis: a systematic review and meta-analysis. Eur J Heart Fail.

[9] Koehler F, Koehler K, Deckwart O, Prescher S, Wegscheider K, Kirwan B, Winkler S, Vettorazzi E, Bruch L, Oeff M, Zugck C, Doerr G, Naegele H, Störk Stefan, Butter C, Sechtem U, Angermann C, Gola G, Prondzinsky R, Edelmann F, Spethmann S, Schellong SM, Schulze PC, Bauersachs J, Wellge B, Schoebel C, Tajsic M, Dreger H, Anker SD, Stangl K (2018). Efficacy of telemedical interventional management in patients with heart failure (TIM-HF2): a randomised, controlled, parallel-group, unmasked trial. Lancet.

[10] Krzesiński P, Jankowska EA, Siebert J, Galas A, Piotrowicz K, Stańczyk A, Siwołowski P, Gutknecht P, Chrom P, Murawski P, Walczak A, Szalewska D, Banasiak W, Ponikowski P, Gielerak G (2022). Effects of an outpatient intervention comprising nurse-led non-invasive assessments, telemedicine support and remote cardiologists' decisions in patients with heart failure (AMULET study): a randomised controlled trial. Eur J Heart Fail.

[11] Bekfani T, Fudim M, Cleland JG, Jorbenadze A, von Haehling Stephan, Lorber A, Rothman AM, Stein K, Abraham WT, Sievert H, Anker SD (2021). A current and future outlook on upcoming technologies in remote monitoring of patients with heart failure. Eur J Heart Fail.

[12] Dierckx R, Inglis SC, Clark RA, Prieto-Merino D, Cleland JG (2017). Telemedicine in heart failure: new insights from the Cochrane meta-analyses. Eur J Heart Fail.

[13] Nguyen HTT, Ha TTT, Tran HB, Nguyen DV, Pham HM, Tran PM, Pham TM, Allison TG, Reid CM, Kirkpatrick JN (2023). Relationship between BMI and prognosis of chronic heart failure outpatients in Vietnam: a single-center study. Front Nutr.

[14] Chow SC, Shao J, Wang H, Lokhnygina Y (2017). Sample Size Calculations in Clinical Research.

[15] Regional Office for the Western Pacific (2000). The Asia-Pacific perspective: redefining obesity and its treatment. World Health Organization.

[16] Nohria A, Tsang SW, Fang JC, Lewis EF, Jarcho JA, Mudge GH, Stevenson LW (2003). Clinical assessment identifies hemodynamic profiles that predict outcomes in patients admitted with heart failure. J Am Coll Cardiol.

[17] Emmons-Bell S, Johnson C, Roth G (2022). Prevalence, incidence and survival of heart failure: a systematic review. Heart.

[18] Bozkurt B, Ahmad T, Alexander KM, Baker WL, Bosak K, Breathett K, Fonarow GC, Heidenreich P, Ho JE, Hsich E, Ibrahim NE, Jones LM, Khan SS, Khazanie P, Koelling T, Krumholz HM, Khush KK, Lee C, Morris AA, Page RL, Pandey A, Piano MR, Stehlik J, Stevenson LW, Teerlink JR, Vaduganathan M, Ziaeian B, Writing Committee Members (2023). Heart failure epidemiology and outcomes statistics: a report of the Heart Failure Society of America. J Card Fail.

[19] Zile MR, Bennett TD, St. John Sutton M, Cho YK, Adamson PB, Aaron MF, Aranda JM, Abraham WT, Smart FW, Stevenson LW, Kueffer FJ, Bourge RC (2008). Transition from chronic compensated to acute decompensated heart failure. Circulation.

[20] Adamson PB (2009). Pathophysiology of the transition from chronic compensated and acute decompensated heart failure: new insights from continuous monitoring devices. Curr Heart Fail Rep.

[21] Punnoose LR, Gopal DM, Stevenson LW (2019). Time to update our profiles. Eur J Heart Fail.

[22] Piskulic D, McDermott S, Seal L, Vallaire S, Norris CM (2021). Virtual visits in cardiovascular disease: a rapid review of the evidence. Eur J Cardiovasc Nurs.

[23] Ploux S, Strik M, Ramirez FD, Buliard S, Chauvel R, Dos Santos P, Haïssaguerre Michel, Jobbé-Duval Antoine, Picard F, Riocreux C, Eschalier R, Bordachar P (2023). Remote management of worsening heart failure to avoid hospitalization in a real-world setting. ESC Heart Fail.

[24] Kuan PX, Chan WK, Fern Ying DK, Rahman MAA, Peariasamy KM, Lai NM, Mills NL, Anand A (2022). Efficacy of telemedicine for the management of cardiovascular disease: a systematic review and meta-analysis. The Lancet Digital Health.

[25] Takeda A, Martin N, Taylor RS, Taylor SJ (2019). Disease management interventions for heart failure. Cochrane Database Syst Rev.

[26] Eberly LA, Tennison A, Mays D, Hsu C, Yang C, Benally E, Beyuka H, Feliciano B, Norman CJ, Brueckner MY, Bowannie C, Schwartz DR, Lindsey E, Friedman S, Ketner E, Detsoi-Smiley P, Shyr Y, Shin S, Merino M (2024). Telephone-based guideline-directed medical therapy optimization in Navajo nation: the Hózhó randomized clinical trial. JAMA Intern Med.

[27] Takahashi EA, Schwamm LH, Adeoye OM, Alabi O, Jahangir E, Misra S, Still CH, American Heart Association Council on Cardiovascular RadiologyIntervention‚ Council on Hypertension‚ Council on the Kidney in Cardiovascular Disease‚Stroke Council (2022). An overview of telehealth in the management of cardiovascular disease: a scientific statement from the American Heart Association. Circulation.

