# Supplementary figures and images for: Effect of a Telemedicine Model on Patients With Heart Failure With Reduced Ejection Fraction in a Resource-Limited Setting in Vietnam: Cohort Study
Source: J Med Internet Res. 2025 Mar 19;27:e67228. doi: 10.2196/67228 (PMC11966076; doi:10.2196/67228)

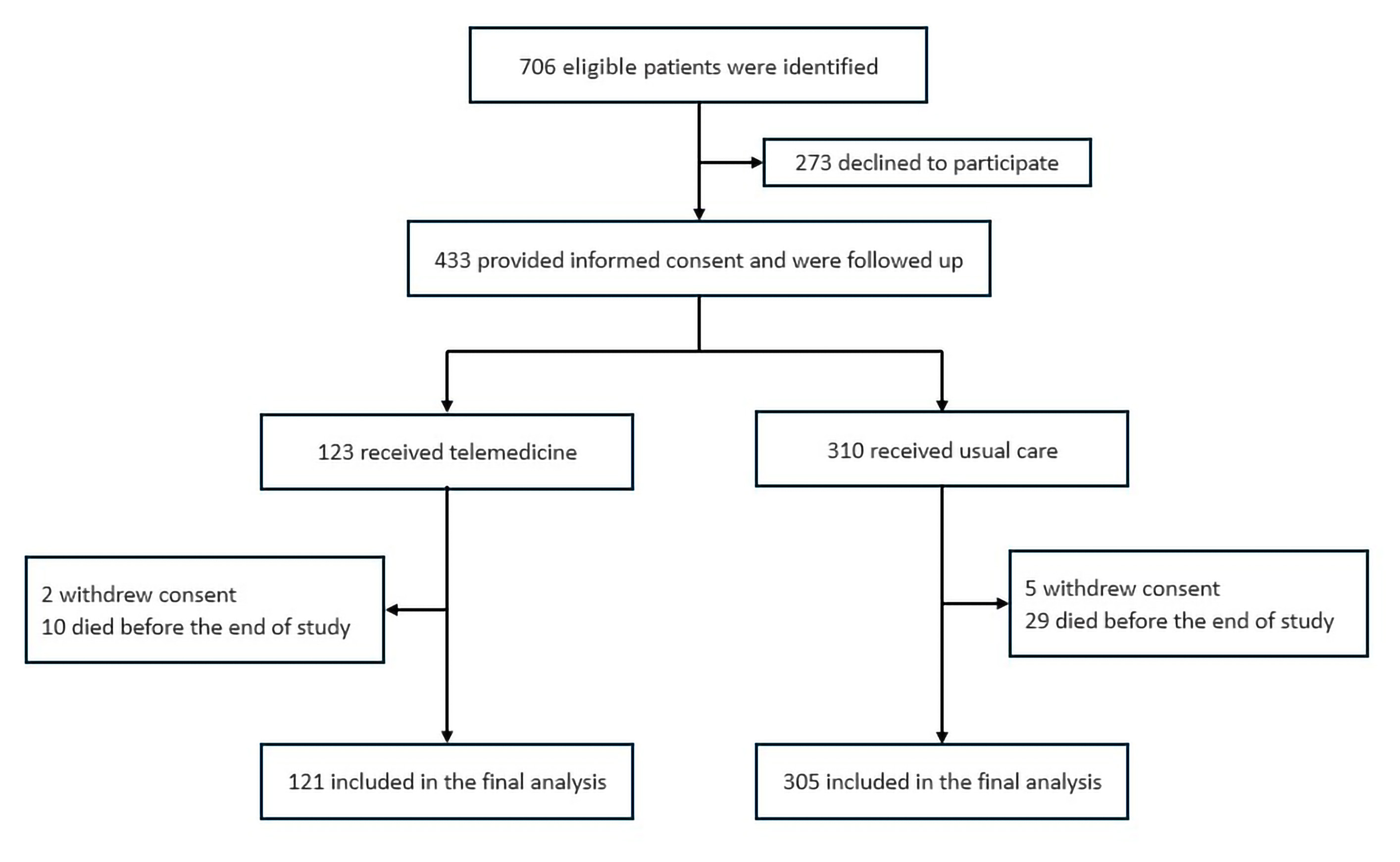

Supplement: Multimedia Appendix 1 [file jmir_v27i1e67228_app1.png]
