# Supplementary material for: Effect of a Telemedicine Model on Patients With Heart Failure With Reduced Ejection Fraction in a Resource-Limited Setting in Vietnam: Cohort Study
Source: J Med Internet Res. 2025 Mar 19;27:e67228. doi: 10.2196/67228 (PMC11966076; doi:10.2196/67228)
Supplement: Multimedia Appendix 2 [file jmir_v27i1e67228_app2.pdf]

I. Sensitivity analysis

Table 1. Multivariable Cox proportional hazards regression assessing the effect of telemedicine on outcomes, including pre-specified confounding variables and additional factors

	Primary outcome		All-cause Mortality		HF Hospitalization	
	aHR [95% CI]	*P*	aHR (95% CI)	*P*	aHR (95% CI)	*P*
**Pre-specified variables**						
**Care model**						
Telemedicine	0.56 [0.34–0.93]	.025	0.82 [0.37-1.81]	.629	0.55 [0.32-0.97]	.037
Usual care	1.0 (reference)	–	1.0 (reference)	–	1.0 (reference)	–
Age (years)	1.01 [0.98-1.03]	.379	1.04 [0.99-1.08]	.113	1.02 [0.99-1.05]	.235
**Sex**						
Male	1.32 [0.82-2.14]	.258	1.01 [0.49-2.10]	.976	1.40 [0.82-2.40]	.214
Female	1.0 (reference)	–	1.0 (reference)	–	1.0 (reference)	–
LVEF (%)	0.92 [0.89-0.96]	<0.01	0.97 [0.91-1.03]	.348	0.91 [0.87-0.95]	<0.01
**BMI classification**						
Underweight	1.14 [0.59-2.19]	.693	1.16 [0.45-3.03]	.758	1.13 [0.55-2.33]	.742
Normal weight	1.0 (reference)	–	1.0 (reference)	–	1.0 (reference)	–
Overweight	0.77 [0.42-1.38]	.376	0.86 [0.30-2.41]	.768	0.89 [0.48-1.66]	.710
Obesity	1.26 [0.69-2.29]	.457	1.16 [0.40-3.33]	.783	1.37 [0.70-2.68]	.353
Hypertension	0.66 [0.40-1.10]	.112	0.44 [0.17-1.10]	.078	0.78 [0.45-1.34]	.368
Diabetes	1.90 [1.17–3.09]	.010	0.98 [0.41-2.33]	.958	1.88 [1.11-3.16]	.018
CAD	1.26 [0.78-2.04]	.348	0.99 [0.43-2.27]	.974	1.54 [0.93-2.55]	.095
CKD	1.47 [0.87-2.49]	.153	1.49 [0.63-3.55]	.364	1.77 [1.01-3.09]	.046
Prior stroke	2.16 [1.04-4.47]	.039	3.80 [1.23-11.72]	.020	1.72 [0.77-3.85]	.189
Atrial fibrillation	1.06 [0.57-1.95]	.863	1.70 [0.72-4.02]	.226	1.11 [0.57-2.17]	.766
NT–proBNP (per 200 ng/mL)	1.00 [0.99-1.01]	.309	1.00 [0.99-1.01]	.880	1.00 [0.99-1.01]	.211
**Medications use**						
ARNI/ACEI/ARB						
Not use	1.0 (reference)	–	1.0 (reference)	–	1.0 (reference)	–
ACEIs/ARBs	1.74 [0.84-3.58]	.133	1.05 [0.31-3.58]	.941	1.53 [0.71-3.30]	.276
ARNI	1.75 [0.80-3.82]	.162	1.46 [0.40-5.28]	.564	1.67 [0.74-3.80]	.220
SGLT2 inhibitors	0.99 [0.55-1.76]	.966	0.69 [0.27-1.79]	.445	1.09 [0.57-2.07]	.798
Beta-blockers	0.60 [0.36-0.98]	.043	0.46 [0.20-1.07]	.071	0.60 [0.35-1.03]	.065
MRAs	0.77 [0.42-1.41]	.393	1.42 [0.49-4.12]	.523	0.78 [0.41-1.51]	.462

Loop diuretics	1.02 [0.59-1.76]	.945	1.25 [0.48-3.27]	.643	0.91 [0.51-1.63]	.752
**Additional Factors**						
**Additional cardiac comorbidities**						
Documented VT	1.03 [0.50-2.15]	.935	1.45 [0.56-3.76]	.440	0.94 [0.41-2.15]	.882
Diagnosed VHD	0.99 [0.63-1.56]	.964	1.11 [0.51-2.42]	.798	0.86 [0.52-1.42]	.554
Diagnosed CM	1.32 [0.85-2.03]	.212	1.42 [0.71-2.86]	.324	1.20 [0.74-1.94]	.459
**Additional relevant medications**						
Thiazide-like diuretics	0.83 [0.35-1.96]	.668	1.01 [0.29-3.53]	.988	0.88 [0.34-2.28]	.796
Ivabradine	1.04 [0.63-1.72]	.873	1.02 [0.44-2.35]	.969	1.09 [0.63-1.88]	.769
**Locality**						
Rural	1.0 (reference)	–	1.0 (reference)	–	1.0 (reference)	–
Urban	0.78 [0.52-1.20]	.259	0.95 [0.47-1.89]	.879	0.88 [0.56-1.38]	.581
**Marital status**						
Never married	1.0 (reference)	–	1.0 (reference)	–	1.0 (reference)	–
Married	0.41 [0.11-1.62]	.206	0.26 [0.02-2.79]	.267	0.41 [0.08-2.07]	.282
Widowed/divorced	0.48 [0.09-2.57]	.395	0.77 [0.05-11.06]	.851	0.43 [0.06-2.99]	.393
**Education**						
School	1.0 (reference)	–	1.0 (reference)	–	1.0 (reference)	–
College/University	1.29 [0.72-2.30]	.390	1.74 [0.63-4.84]	.286	1.37 [0.72-2.61]	.333
**Monthly income**						
High (> 450 USD)	0.57 [0.28-1.13]	.111	0.78 [0.25-2.46]	.672	0.57 [0.26-1.23]	.150
Medium (300 – 450 USD)	1.0 (reference)	–	1.0 (reference)	–	1.0 (reference)	–
**Monthly treatment affordability**						
High (> 125 USD)	1.46 [0.83-2.56]	.191	1.29 [0.53-3.12]	.574	1.62 [0.87-3.03]	.128
Medium (75 – 125 USD)	1.0 (reference)	–	1.0 (reference)	–	1.0 (reference)	–

aHR: adjusted Hazard ratio; CI: confidence interval; HF: heart failure; LVEF: left ventricular ejection fraction; CAD: coronary artery disease; CKD: chronic kidney disease; ARNI: angiotensin–neprilysin Inhibitor; ACEIs: angiotensin-converting enzyme inhibitors; ARBs: angiotensin receptor blockers; SGLT2: Sodium-glucose cotransporter-2; MRAs: mineralocorticoid receptor antagonist; NT-proBNP: N-terminal pro b-type natriuretic peptide; VT: ventricular tachycardia; VHD: valvular heart disease; CM: cardiomyopathy.
